# Mini-SEA: Validity and Normative Data for the French-Quebec Population Aged 50 Years and Above

**DOI:** 10.1093/arclin/acae051

**Published:** 2024-06-25

**Authors:** Hannah Mulet-Perreault, Mariane Landry, Robert Jr Laforce, Joël Macoir, Carol Hudon

**Affiliations:** École de psychologie, Faculté des sciences sociales, Université Laval, Québec, QC, Canada; Centre de recherche CERVO, Centre intégré universitaire de santé et de services sociaux de la Capitale-Nationale, Québec, QC, Canada; École de psychologie, Faculté des sciences sociales, Université Laval, Québec, QC, Canada; Centre de recherche CERVO, Centre intégré universitaire de santé et de services sociaux de la Capitale-Nationale, Québec, QC, Canada; Clinique Interdisciplinaire de Mémoire, CHU de Québec-Université Laval, Québec, QC, Canada; Département de médecine, Faculté de Médecine, Université Laval, Québec, QC, Canada; Centre de recherche CERVO, Centre intégré universitaire de santé et de services sociaux de la Capitale-Nationale, Québec, QC, Canada; École des sciences de la réadaptation, Faculté de Médecine, Université Laval, Québec, QC, Canada; École de psychologie, Faculté des sciences sociales, Université Laval, Québec, QC, Canada; Centre de recherche CERVO, Centre intégré universitaire de santé et de services sociaux de la Capitale-Nationale, Québec, QC, Canada; Centre de recherche VITAM, Centre intégré universitaire de santé et de services sociaux de la Capitale-Nationale, Québec, QC, Canada

**Keywords:** Mini-SEA, Social cognition, Mild cognitive impairment, Alzheimer’s disease, Normative data, Validity

## Abstract

**Objective:**

The mini Social cognition & Emotional Assessment (mini-SEA) is a social cognition battery which assesses theory of mind and emotion recognition. Currently, no psychometrically validated measure of social cognition with adapted normative data exists for the middle-aged and elderly French-Quebec population. This project aims to determine the known-group discriminant validity of a cultural and linguistic adaptation of the mini-SEA between cognitively healthy people, those with mild cognitive impairment (MCI) or living with Alzheimer’s Disease (AD). This study also aims to examine the stability of mini-SEA’s performance over a 3–4-month time period, as well as to produce normative data for French-Quebec people aged 50 years. Normative data are derived for the full and an abbreviated version of the Faux Pas subtest.

**Method:**

The sample included 211 French-speaking participants from Quebec (Canada) aged 50 to 89 years. Mini-SEA’s performance between a sub-sample of cognitively healthy people (*n* = 20), those with MCI (*n* = 20) or with AD (*n* = 20) was compared. A sub-sample of cognitively healthy people (*n* = 30) performed the task twice to estimate test–retest reliability. Socio-demographic variables’ effects on scores were examined to produce normative data in the form of regression equations or percentile ranks.

**Results:**

Significant differences emerged between cognitively healthy people and those with MCI or AD. Moreover, scores were relatively stable over a period of 3 to 4 months. Finally, for the normative data, age, gender, and education were associated with performance on the mini-SEA or its subtests.

**Conclusions:**

This study improves and standardizes social cognition’s assessment among French–Quebec individuals, which will help characterize their cognitive profile.

## INTRODUCTION

The proportion of seniors in Quebec (Canada) is rapidly increasing. It is estimated that the number of people aged 65 and above will rise by nearly 1.1 million from 2020 to 2066. Thus, the proportion of the elderly population within the total population will increase from 20% to 27% (Institut de la statistique du Québec [ISQ], 2021). Considering that the risk of developing a neurocognitive disorder increases with age, doubling every 5 years from the age of 64, it is expected that the number of people with dementia will increase significantly in line with demographic aging (Public Health Agency, 2017). Early diagnosis of neurocognitive disorders can help alleviate the consequences of this class of diseases in several ways (Rasmussen & Langerman, 2019). Numerous diagnostic tools are currently available to assess the cognitive functions affected early in the dementia process (e.g., memory, language, executive functions; Carrier et al., 2022). Nevertheless, few tests exist to measure social cognition.

Social cognition refers to the cognitive processes involved in interpersonal relationships (McDonald & Kumfor, 2022). It implicates several functions including low-level (e.g., perception and recognition of emotions) and high-level (e.g., understanding other’s mental states and behaviors) cognitive skills (Speranza, 2009). The theory of mind (ToM) is one of many components of social cognition and it is known as the ability to infer the intentions, thoughts, or feelings of others. ToM is subdivided into a cognitive and an affective component, respectively referring to attributions regarding cognitive mental states such as beliefs and intentions, and to attributions about emotional mental states (Ruitenberg et al., 2020).

Social cognition is essential for having social interactions and behaviors considered normal (Bertoux & Hornberger, 2015). However, this function is affected in several age-associated neurocognitive disorders (Osborne-Crowley et al., 2022), particularly in behavioral variant of frontotemporal dementia (Park et al., 2017), dementia with Lewy bodies (Heitz et al., 2016), and Alzheimer’s disease (AD; Klein-Koerkamp et al., 2012) and its prodromal stage, namely mild cognitive impairment (MCI; Gaudreau et al., 2015; McCade et al., 2011). Focussing on AD, evidence shows significant deficits in social cognition in this neurocognitive disorder, particularly with regard to ToM, but also emotion recognition (Irish & Ramanan, 2021; Klein-Koerkamp et al., 2012; Schild et al., 2021; Setién-Suero et al., 2022; Wong & Kumfor, 2022). To a lesser extent than in AD, difficulties in the ToM and emotion recognition are also found in people living with MCI, when compared with cognitively healthy older people (Bora & Yener, 2017; Morellini et al., 2022a; Yi et al., 2020).

In healthy cognitive aging, there is a progressive deterioration of some processes involved in social cognition, but to a lesser extent than in neurocognitive disorders (Fernandes et al., 2021). Several authors have reported a decrease in performance in tests evaluating ToM with advancing age (Baksh et al., 2018; Bottiroli et al., 2016; Cavallini et al., 2013). Indeed, it has been shown that performance in cognitive ToM is negatively associated with age, but that performance in affective ToM remains rather stable in normal aging (Bottiroli et al., 2016; Reiter et al., 2017). In addition, aging affects other processes such as the ability to recognize emotions (Ferreira et al., 2021; Quesque et al., 2022). More specifically, some authors noted that as people age, recognition of negative emotions is more altered than recognition of emotions in general (Keightley et al., 2006; Kemp et al., 2012). For instance, emotions such as fear or sadness are recognized with less accuracy than joy or surprise during normal cognitive aging.

The mini-SEA (Bertoux et al., 2013) is a social cognition battery that consists of two tests: a *Faux Pas* test, which assesses both components of ToM, and a facial emotion recognition task. The test was developed in France and is now used in several countries. Normative data from a recent mini-SEA validation study among the French (France) population (*n* = 150; 18–85 years of age) showed that task performance is influenced by sociodemographic factors. More precisely, increasing age was negatively associated with mini-SEA’s performance and women performed slightly better than men in this battery. However, education was not associated with mini-SEA’s performance (Quesque et al., 2020).

For a standardized interpretation of an individual’s performance in a test, neuropsychologists must compare their patient’s results to a normative sample in order to position their score in the corresponding reference group within the population (Amieva, 2016). As a result, the performance of examinees can be compared to what would be expected according to their age, sex, and level of education. Currently, clinicians report a glaring lack of normative data for several neuropsychological tests (Branco Lopes et al., 2021; Monette et al., 2023). This is particularly true for tools assessing social cognition since it is a cognitive domain for which applied research has developed rather recently (Quesque et al., 2020). Adequate standardized assessment of social cognition in older adults with suspected cognitive decline is crucial for several reasons. First, it is recognized that abnormal decoding of social information is associated with the progression from MCI to AD (Bediou et al., 2009), and also this function is impaired in several neurocognitive disorders as mentioned above. Second, deficits in social cognition may negatively influence several spheres of the individual’s life for which there are opportunities for intervention (Klein-Koerkamp et al., 2012), such as preventive measures for family members, that address the potential impact of deficits in social cognition, such as inappropriate behavior, misjudging situations, misunderstanding social cues, increased risk of financial abuse, and increased burden on family caregivers (Ellison, 2021; Han et al., 2016; Hargrave et al., 2002; Martinez et al., 2018; Morese & Palermo, 2022; Shimokawa et al., 2001; Spitzer et al., 2019; Torres et al., 2015). Currently, no social cognition tests in Quebec (Canada) have been validated or standardized for the population aged 50 and above (Carrier et al., 2022). In addition, according to a recent study comparing performance on mini-SEA between 12 different countries, including one from Quebec (Quesque et al., 2022), significant differences were found in performances on a social cognition test depending on the examinee’s culture. This further confirms the need to validate and obtain local norms for social cognition assessment tools.

Thereby, this study generally aims to evaluate a set of psychometric properties of the mini-SEA and to develop normative data for this battery among French–Quebec individuals aged 50 years and above. More specifically, the first specific objective is to estimate the known-group discriminant validity of the mini-SEA between cognitively healthy individuals and those living with MCI or AD. The second specific objective is to verify the stability of performances over time (test–retest reliability) in the cognitively healthy group. Finally, the third specific objective is to produce normative data adapted to the French-Quebec population for the mini-SEA and its subtests, but also for a short version of the Faux Pas Test (short-FPT).

## MATERIALS AND METHODS

### Participants

The final sample comprised 211 participants aged between 50 and 89 years (171 cognitively healthy people, 20 people living with MCI, and 20 people living with AD). To be included, participants had to have completed at least 6 years of schooling so that they could read the mini-SEA’s stories of *Faux Pas* without difficulty. The number of years of schooling was a continuous variable but for reference, they were calculated based on the Quebec education system (Éducation internationale, n.d.; Gouvernement du Québec, 2023), where 6 years represent elementary school, 11 years a high school diploma, 13 years a college diploma (14 years a college/technical program), 16 years a bachelor’s degree, 18 years a master’s degree, 21 years a doctorate, and 23 years a medical or postdoctoral fellowship. Participants were all recruited from the database of potential participants of Hudon and Macoir’s laboratories at the CERVO Brain Research Centre. This database is continually updated through different means (e.g., ads on the research center’s website, on an email distribution list of Université Laval, and at various public events such as the local Alzheimer’s Awareness Day). About 40% of both MCI and AD participants were recruited at a tertiary memory clinic (La Clinique Interdisciplinaire de Mémoire du CHU de Québec, Hôpital de l’Enfant-Jésus, Québec City, Canada).

Participants in the cognitively healthy control group were selected based on whether they had no cognitive complaints, as defined by the criteria for subjective cognitive decline (Jessen et al., 2020). Cognitively healthy individuals also needed to have a Z score on the MoCA (7.1 version in French) higher or equal to −1.50 (Larouche et al., 2016; Nasreddine et al., 2005). Participants in the MCI group met the criteria for MCI of Albert et al. (2011). Participants in the AD group were recruited based on whether they have been given a probable diagnosis of AD by a physician. They also needed to meet McKhann et al.’ (2011) criteria for AD. All study procedures have been approved by the Research Ethics Board on neuroscience and mental health of the *Centre intégré universitaire de santé et de services sociaux de la Capitale-Nationale* (CIUSSS-CN, approval #2019-1541).

Participants were excluded if they presented one or more of the following criteria: (1) history of moderate or severe head trauma; 2) history of stroke; 3) history of delirium in the past 6 months; 4) history of intracranial surgery; 5) brain neurological disorders (except for MCI or AD); 6) history of bacterial encephalitis or meningitis; 7) untreated medical or metabolic condition; 8) history of psychotic symptoms or manic episode(s); 9) oncological treatments in the last 12 months; 10) alcoholism or substance abuse; 11) general anesthesia in the last six months; 12) uncorrected vision or hearing problems. All exclusion criteria were self-reported. An additional objective exclusion criterion was considered for the normative data portion of the study. Namely, participants had to get a Z-score on the MoCA equals or above −1.50.

### Materials

All materials in this study were in French. A socio-demographic questionnaire was completed with each participant to collect information such as age, gender, number of years of education, first and usual language, etc. The MoCA was also used with all control participants as a cognitive screening tool (Larouche et al., 2016; Nasreddine et al., 2005). For participants with a diagnosis of AD or MCI and for those whose potential cognitive decline had been screened using the MoCA, then a neuropsychological evaluation of approximately two hours was carried out (see Table 1 for details of the questionnaires and tests administered).

**Table 1:** List of the questionnaires and tests administered in the brief neuropsychological evaluation

	**Test/Questionnaire**	**Clinical/Cognitive measures**	**References**
1	History interview	Information about the participants	Hudon (unpublished)
2	MoCA	Global cognitive functioning	Larouche et al. (2016); Nasreddine et al. (2005)
3	Subjective Cognitive Decline Questionnaire	Cognitive complaint	Jessen et al. (2020)
4	16-item Free Recall/Cued Recall Test	Verbal episodic memory	Dion et al. (2015); Van der Linden et al. (2004)
5	Trail-Making A/B	Executive functions	Reitan (1979); St-Hilaire et al. (2018)
6	Rey-Osterrieth Complex Figure	Immediate visual episodic memory/Visuoconstructive skills/Executive functions	Osterrieth (1944); Rey (1941); Tremblay et al. (2015)
7	Size-match task (BORB)	Visual perception	Riddoch and Humphreys (1993)
8	Victoria Stroop test	Executive functions	Regard (1981); Tremblay et al. (2016)
9	Code subtest (WAIS-IV)	Information processing speed	Wechsler (2008)
10	Phonemic (T-N-P) and semantic (animals) verbal fluency	Language/Executive functions	Consortium of Montreal and McGill University (1996); St-Hilaire et al. (2016)
11	TDQ-30 (confrontation naming test)	Language	Macoir et al. (2021)
12	Pyramids and Palms Trees Test	Semantic memory	Callahan et al. (2010); Howard & Pattersen (1992)
13	Activities of Daily Living Inventory	Functional autonomy	Galasko et al. (2006)
14	30-item Geriatric Depression Scale	Depressive symptoms	Bourque et al. (1990); Yesavage (1988)

*Note*. *MoCA* = Montreal Cognitive Assessment; *BORB* = Birmingham Object Recognition Battery; *WAIS-IV* = Wechsler Adult Intelligence Scale 4^th^ Edition; *TDQ-30* = Test de dénomination de Québec 30 items.

To be used in Quebec, the mini-SEA had to be adapted due to cultural and linguistic specificities. Indeed, although the battery was already in French initially, some terms or names used in the original mini-SEA were not common in Quebec or did not have the same meaning. Five minor modifications were made. For example, the term “football” was replaced by “soccer” to reflect the lexical-semantic reality in Quebec, where the first term refers to a different sport (story #0). Another modification consisted in changing “dinner” (dîner) to “supper” (souper) (story #7), reflecting the linguistic differences between France and Quebec. Additionally, the abbreviation “m’dam” was adjusted for “madam”, as it is not commonly used in Quebec (story #10). The name “Céline” was substituted with “Sarah” due to “Céline” being perceived as an “old” name for a three-year-old child in Québec (story #4). Lastly, the English quotation marks were replaced with French quotations marks. The French-Quebec adaptation of the mini-SEA is based on the most recent version, dating from 2014. This battery includes two social cognition tasks: a modified and reduced version of the Faux Pas Test (FPT; Stone et al., 1998) as well as Facial Emotion Recognition Test (FERT) which is a reduced version of the Ekman 60 Faces Test (Ekman & Friesen, 1975).

The FPT subtest, which assesses ToM, contains 10 written short stories illustrated with pictures. Some stories include awkward situations, so-called Faux Pas, and others do not (Bertoux et al., 2013). The questions measure the ability to decode social rules and infer the intentions of others through socially awkward situations. Control questions are also asked to ensure that the person has understood the different stories relatively well. The FPT score is out of 40 points: 30 points are awarded for the Faux Pas stories and 10 points for the stories without faux pas. The FPT score is then reported on 15 points using a cross-multiplication procedure. Note that the short-FPT was developed as part of this project to facilitate its use in clinical settings. This version comprises five stories (two without a faux pas story and three with a faux pas story) and is scored out of a total of 22 points. The rationale for the selection of the stories in the short-FPT is explained in the Statistical analyses’ section.

The FERT subtest includes 35 pictures of faces representing an emotion. The different possible emotions are joy, surprise, sadness, disgust, anger, fear, and neutrality. For each face, the examinee must specify in less than 12 s which emotion is depicted. The seven choices of emotions are written below every picture. The score is out of 35 but is then converted to out of 15 points also using a cross-multiplication procedure.

The total score for the mini-SEA is out of 30, which refers to the sum of the scores for the FPT and FERT subtests. For its administration, it is necessary to have the scoring sheet and the stimuli for the participant, which includes the Faux Pas stories and the 35 pictures for the FERT (Bertoux et al., 2013). For this study, an electronic tablet was used to present the stimuli to the participants. It takes about 30 min to administer the mini-SEA. Interested clinicians may request the scoring sheet, stimuli as well as the administration and scoring guide from the corresponding author.

### Procedure

This study is part of a larger validation and normative study of several cognitive and language tests. Most of the participants in the cognitively healthy control group attended two assessment sessions of approximately 90 min at the CERVO Brain Research Centre. During the first session, the research team obtained written consent and filled out a socio-demographic form. Then, the MoCA was administered to ensure that the control participants did not present any cognitive impairment. The mini-SEA was administered during the first session as well as other tests assessing psychomotor speed (Grooved Pegboard Test; Lafayette Instrument, 2023), verbal fluency (verbs, Macoir & Hudon, 2023a; constraint, Macoir & Hudon, 2023b; famous people, Macoir & Hudon, unpublished), visual episodic memory (Location Learning Test; Bucks & Willison, 1997), inhibition (Hayling’s test; Burgess & Shallice, 1997), and language (Naming action verbs – DVAQ-30 test; Macoir et al., 2023). The order in which the tests were administered was pre-established to avoid any possible interference effect between the different tasks and participants were assessed by trained research assistants. In the second session, other tests of the protocol were administered.

A subsample of 30 cognitively healthy control participants, who had already agreed to be contacted again in a few months to retake certain tests, was also seen again 3 to 4 months later to retake the mini-SEA. Another subsample of 20 cognitively healthy control participants was matched with participants of the MCI and AD groups in terms of age and level of education to assess the known-group discriminant validity of the mini-SEA. Recruitment of control participants took place between 2018 and 2021 (with a pause because of the COVID-19 pandemic).

Participants living with MCI or AD were met three times for this project. First, their cognitive profile was assessed with a brief clinical and neuropsychological evaluation (Table 1) of 2 h to confirm their eligibility for the study and to determine to which group they belonged. A socio-demographic form was also filled out. Following this cognitive evaluation, the participant’s MCI or AD status was confirmed at a consensus meeting that included members of the research team as well as an experienced and licensed neuropsychologist. Then, two assessment sessions of approximately 90 min for the project were carried out. To minimize the risk of interference and potential variations in the procedures compared to the cognitively healthy group, the neuropsychological assessment session for MCI and AD participants was scheduled with a minimum one-week interval before the first of the two experimental sessions. During the second assessment session, participants were administered the mini-SEA and other tests measuring verbal fluency (verbs [Macoir & Hudon, 2023a], constraint and alternating [Macoir & Hudon, 2023b], famous people [Macoir & Hudon, unpublished]), inhibition (Hayling’s test; Burgess & Shallice, 1997), and language (DVAQ-30 test, Macoir et al., 2023; Batterie of rapid naming– BARD, Macoir & Hudon, 2021). As for the control participants, the test administration order was pre-established and carried out by trained research assistants. Recruitment of MCI or AD participants took place between 2019 and 2023 (with a pause because of the COVID-19 pandemic).

### Statistical analyses

Descriptive analyses were first performed using the sociodemographic variables (sex, age, education) and the MoCA score. Sociodemographic variables were all continuous variables except for sex, which was coded 0 for women and 1 for men. For the main analysis, the scores of interests were the FPT score, the FERT score, and the mini-SEA total score. For all three objectives, Shapiro–Wilks and Kolmogorov–Smirnov tests as well as histograms were first performed to determine whether the score distributions were normal.

For the first objective, which was to compare mini-SEA’s performance between control, MCI, and AD groups, an analysis of variance (ANOVA) was carried out for the mini-SEA total score since the distribution of the data was normal. A Kruskal–Wallis test was used for the FPT and the FERT since their score distributions were skewed. The interactions between the groups and the mini-SEA total score and or FPT score were then examined using Tukey post-hoc analysis and Dunn post-hoc analysis to determine between which groups there were differences (healthy control vs MCI; healthy control vs AD; MCI vs AD).

The second objective was to determine whether performance on the mini-SEA remained stable after three to four months in a subsample of 30 control healthy participants. Since the distribution of scores was not normal, Spearman correlations were performed to verify the association between the two administrations of the mini-SEA.

For the normative data, the dependent variables were the three mini-SEA scores described above (FPT score, FERT score, and total score) and the independent variables were the socio-demographic variables (age, education, and sex). Multiple linear regression was performed with the sociodemographic variables for the FERT score and the total mini-SEA score. They were considered predictors in the model if their *p* value was less than .10. Then, a Z-score equation was derived from the standard error of estimate of the multiple regression. Since the FPT score was not normally distributed, percentile ranks were computed instead.

Item-by-item analyses were then performed for each story to create the shorter version of the FPT to facilitate the administration of this subtest in a clinical context with time constraints. To determine which stories to choose among the 10 of the original mini-SEA, Kruskal–Wallis tests were carried out to compare the performance for each story between the cognitively healthy, MCI, and AD groups. The retained stories were those that best discriminated between cognitively healthy participants and those with cognitive impairment. Since the distribution of the scores for the short-FPT was not normal, percentile ranks were computed to produce normative data.

All statistical analyses were performed using IBM SPSS software (version 28) at an alpha level of 0.05.

## RESULTS

The initial sample comprised 223 Quebec residents aged 50 and above with French as their first and usual language. Twelve participants were excluded from the study for the following reasons: Montreal Cognitive Assessment (MoCA; Nasreddine et al., 2005) Z-score below −1.50 for cognitively healthy participants (*n* = 6), withdrawal from study participation (*n* = 2), presence of a severe head trauma (*n* = 1), incorrect group assignment (*n* = 1), and extreme scores (*n* = 2). Compared to the participants included in the study, those excluded were not significantly different in terms of sociodemographic variables (age, sex, education; all *p >* .05), but had lower MoCA scores (*U* = 629.00, *p* = .003). The final sample comprised 211 participants aged between 50 and 89 years (mean = 70.8; SD = 8.0), with an educational level ranging from 7 to 21 years (mean = 14.2; SD = 3.0), and with a female representation of 58.8%. The descriptive analyses of the subsamples included for the three objectives are presented in Tables 2, 3, and 4, respectively.

**Table 2:** Descriptive statistics of participants in the cognitively healthy, mild cognitive impairment, and Alzheimer’s disease groups, and comparison of participants on mini-SEA scores (*n* = 60)

Characteristics	Healthy *n* = 20	MCI *n* = 20	AD *n* = 20	*p*	H *vs.* MCI	H *vs.* AD	MCI *vs.* AD
**Sociodemographic and cognitive screening**							
Age, *mean years* (*SD*)	74.6 (5.0)	76.5 (6.0)	74.2 (7.3)	.453^a^	-	-	-
Education, *mean years* (*SD*)	14.4 (3.7)	13.8 (3.2)	14.4 (3.7)	.906^b^	-	-	-
Sex, female *n* (*%*)	10 (50.0)	11 (55.0)	7 (35.0)	.419^c^	-	-	-
MoCA, *mean Z score* (*SD*) [*Min-Max scores*]	0.3 (0.8) [22–29]	−1.1 (1.3) [17–27]	−3.0 (1.5) [11–24]	< .001^b^	0.004^*^	< 0.001^*^	0.003^*^
**Test performance score**							
FPT score (/40), *mean* (*SD*) [95% CI]	33.9 (4.2) [31.0–36.7]	28.1 (6.6) [25.2–30.9]	25.1 (8.0) [22.2–28.0]	< .001^b^	0.006^*^	< 0.001^*^	0.280
FERT score (/35), *mean* (*SD*) [95% CI]	27.9 (2.3) [26.4–29.3]	27.1 (3.8) [25.6–28.5]	26.4 (3.3) [24.9–27.8]	.269^b^	-	-	-
Mini-SEA total score (/30), *mean* (*SD*) [95% CI]	24.6 (1.7) [23.3–26.0]	22.1 (3.8) [20.8–23.5]	21.1 (3.2) [19.7–22.4]	.002^a^	0.029^*^^*^	0.001^*^^*^	0.533

*Note*. *H* = cognitively healthy; *MCI* = mild cognitive impairment; *AD* = Alzheimer’s Disease.

*SD =* Standard Deviation; *Min* = Minimum; *Max* = Maximum.

*FPT* = Faux Pas Test; *FERT* = Facial Emotion Recognition Test.

95% CI = 95% confidence intervals.

^a^One-way ANOVA; ^b^ Kruskal–Wallis; ^c^ Chi-square.

^*^Significant Dunn post-hoc analysis for Kruskal–Wallis test.

^**^Significant Tukey post-hoc analysis for one-way ANOVA.

**Table 3:** Descriptive statistics and correlations coefficients for the test–retest subsample and scores (*n* = 30)

**Sociodemographic and cognitive screening characteristics**				
Age, mean ± SD [min; max]	68.5 ± 8.3 [50.2; 80.1]			
Sex, n female (%)	17 (56.7%)			
Education, mean ± SD [min; max]	14.9 ± 3.0 [7.0; 21.0]			
MoCA Z score, mean ± SD [min; max] MoCA score, mean ± SD [min; max]	0.23 ± 0.75 [−1.31; 1.22] 27.1 ± 2.1 [22–30]			
**Mini-SEA test–retest correlations**				
	1^st^ testing	Retest	*r*	*p*
FPT score, mean (SD)	32.3 (5.5)	31.8 (7.0)	0.622	< .001^*^
FERT score, mean (SD)	27.4 (2.8)	28.7 (2.8)	0.634	< .001^*^
Mini-SEA total score, mean (SD)	23.8 (2.4)	24.2 (2.8)	0.506	.004^*^

*Note. SD* = Standard Deviation; Min = Minimum; Max = Maximum.

MoCA = Montreal Cognitive Assessment Z-score; *FPT* = Faux Pas Test (/40); *FERT* = Facial Emotion Recognition Test (/35); Mini-SEA total score (/30).

^*^Significant correlations coefficients.

**Table 4:** Distribution of participants in the normative sample (*n* = 171)

Characteristics	N (%)
Age (*Mean ± SD [Min;Max]*)	69.8 ± 7.9 [50.2; 89.6]
50–60	18 (10.5)
61–70	71 (41.5)
71–80	64 (37.4)
81–90	18 (10.5)
Women (n (%))	106 (62.0)
Education (*Mean ± SD [Min;Max]*)	14.2 ± 2.9 [7; 21]
0–5	0 (0.0)
6–10	11 (6.4)
11–15	90 (52.6)
16+	70 (40.9)
Cognitive screening	*Mean ± SD [Min;Max]*
MoCA Z-score MoCA score	0.24 ± 0.77 [−1.50; 2.04] 26.9 ± 2.1 [20–30]

*Note. SD* = Standard Deviation; *Min* = Minimum; *Max* = Maximum.

*MoCA* = Montreal Cognitive Assessment.

### Objective 1: Known-group discriminant validity

Table 2 shows the sociodemographic and cognitive screening characteristics of the three groups in the known-group discriminant validity sample. The overall proportion of women and men in the three groups was proportional, and participants in each group were of similar age. Additionally, MoCA Z scores showed greater deficits in the AD group compared to the MCI group and compared to the cognitively healthy group, which was expected.

Table 2 presents the results of the group comparisons for the mini-SEA, FPT, and FERT. Significant differences were found between the groups for the mini-SEA total score (F(2, 57) = 7.266, *p* = .002, η_p_^2^ = 0.203) and the FPT (H(2, 60) = 15.497, *p* < .001, ${E}_H^2$ = 0.263), but not for the FERT (H(2, 60) = 2.623, *p* = 0.269, ${E}_H^2$ = 0.044). Specifically, compared to the healthy control group, the AD and the MCI groups had significantly lower scores on both the mini-SEA total score and the FPT. No significant differences were found between the MCI and AD groups for the total mini-SEA score or its subtests.

### Objective 2: Test–retest reliability

The test–retest reliability of the mini-SEA was assessed by administering the test twice to a sample of 30 cognitively healthy individuals (see Table 3 for sociodemographic and cognitive screening characteristics) approximately within a three- to four-month interval (M = 3.7 months; SD = 0.9; Min = 2.7; Max = 5.8). Note that the correlation coefficients were similar between shorter and longer retest times. Results from Spearman correlations indicate significant test–retest reliability for the mini-SEA total score and for the FPT and FERT scores (see Table 3).

### Objective 3: Normative data

In the cognitively healthy sample, data were missing for the FPT (*n* = 3) and FERT (*n* = 4), which also led to missing data for the mini-SEA total score (*n* = 7). Thus, from the total sample of 171 participants, the following analyses were carried out with 167 participants for the FERT, 168 for the FPT and, finally, 164 for the total score of the mini-SEA. Moreover, three participants had one extreme value among the three scores, which was replaced (or imputed) from the mean.

Sociodemographic and cognitive screening characteristics of the normative sample are presented in Table 4. The sample showed an overrepresentation of women and an elevated proportion of participants with higher education levels when compared to the actual Quebec demographics in Table 5 (ISQ, 2016; ISQ, 2022).

**Table 5:** Comparison of demographic data between the province of Quebec (%) and the study sample (number and %) and (*n* = 171)

	Province of Quebec		Study sample	
	Men	Women	Men	Women
**Age and sex**				
50–64 years	50.20%	49.80%	13 (31.7%)	28 (68.3%)
65–74 years	48.90%	51.10%	34 (43.0%)	45 (57.0%)
75–84 years	46.30%	53.70%	28 (41.9%)	25 (58.1%)
85+ years	36.50%	63.50%	0 (0.0%)	8 (100.0%)
**Education and sex**				
0–12 years	63.20%^a^	60.80%^a^	13 (20.0%)	45 (42.5%)
13+ years	36.80%^a^	39.20%^a^	52 (80.0%)	61 (57.5%)
**Age and education**	*0–12 years*	*13+ years*	*0–12 years*	*13+ years*
45–54 years	52.70%	47.30%	2 (66.7%)^b^	1 (33.3%)^b^
55–64 years	61.90%	38.10%	12 (31.6%)	26 (68.4%)
65–74 years	65.20%	34.80%	25 (31.6%)	54 (68.4%)
75+ years	77.50%	22.50%	19 (37.3%)	32 (62.7%)

^a^For the province of Quebec, the categories also include men and women of 45–49 years.

^b^For the study sample, 50–54 years.

Table 6 reports the descriptive statistics for the total score and subtests scores of the mini-SEA. The descriptives statistics for the short-FPT are also reported. Based on the analyses described in the Method section, the short-FPT was composed of stories #1, #3, #4, #5 and #8 of the long FPT. Stories without faux pas (i.e., #1, #5) were selected because they discriminated cognitively healthy participants from those living with AD and stories with faux pas (i.e., #3, #4, #8) were selected since they best allowed to discriminate cognitively healthy people from those living with MCI or AD (see Table 10 in Supplementary material for statistical results of these analyses).

**Table 6:** Descriptive statistics of the mini-SEA scores for the normative sample

	*Mean*	*SD*	*Min; Max*
**Mini-SEA (n = 164)**			
Mini-SEA total score (/30)	24.28	2.02	19.02–28.39
**FPT (n = 168)**			
FPT total score (/40)	32.53	4.27	21; 40
Stories with faux pas (/30)	22.49	4.99	4; 30
Stories without faux pas (/10)	9.64	0.99	4; 10
Control questions (/20)	19.76	0.49	17; 20
**Short-FPT (n = 168)**			
Short-FPT total score (/22)	18.78	2.814	7; 22
Stories with faux pas (/18)	14.96	8.09	3;18
Stories without faux pas (/4)	3.82	0.33	2; 4
Control questions (/10)	9.92	0.29	8; 12
**FERT (n = 167)**			
FERT total score (/35)	28.41	2.73	20; 35
Joy (/5)	4.95	0.23	4; 5
Anger (/5)	3.96	1.01	0; 5
Fear (/5)	2.14	1.35	0; 5
Disgust (/5)	4.13	0.85	1; 5
Surprise (/5)	4.51	0.72	1; 5
Sadness (/5)	3.64	1.37	0; 5
Neutrality (/5)	4.82	0.44	3; 5

*Note. SD* = Standard Deviation; *Min* = Minimum; *Max* = Maximum.

*FPT* = Faux Pas Test (/40); *short-FPT* = short Faux Pas Test (/22); *FERT* = Facial Emotion Recognition Test (/35).

The regression coefficients and intercepts for each sociodemographic variable by mini-SEA total score and FERT are presented in Table 7. The analyses revealed that the regression model for the FERT was significant (R^2^ = 0.108, F(3, 163) = 6.565, *p* < .001) and the three predictor variables (age, education, and sex) were significantly associated with performance on this subtest. The regression model was also significant for the mini-SEA total score, (R^2^ = 0.050, F(3, 160) = 2.823, *p* = .041), with education being a predictor, while age and sex were marginally significant. The models accounted 10.8% of the variance in FERT score and 5% in the mini-SEA total score. Based on the results obtained from the regression models, Table 8 provides equations to calculate Z-scores for the FERT subtest and the total score of the mini-SEA, taking into account the effects of age, education, and sex.

**Table 7:** Coefficients for multiple linear regression analyses for the Facial Emotion Recognition Test (*n* = 167) and Mini-SEA total scores (*n* = 164)

Variables	*B*	**β**	*T*	*p*
**Facial Emotion Recognition Test** ^a^				
Age	−0.058	−0.168	−2.269	.025^*^
Education	0.185	0.195	2.552	.012^*^
Sex	−1.518	−0.266	−3.483	.001^*^
**Mini-SEA total** ^b^				
Age	−0.035	−0.140	−1.811	.072^*^^*^
Education	0.110	0.159	2.007	.046^*^
Sex	−0.541	−0.129	−1.622	.107^*^^*^

^a^Intercept = 30.344; Square root of the mean square residual = 2.625.

^b^Intercept = 25.373; Square root of the mean square residual = 1.985.

^*^Significant correlations coefficients.

^**^Marginally significant correlations coefficients.

**Table 8:** Regression equation to calculate Z scores for each independent variables on the Facial Emotion Recognition Test (*n* = 167) and Mini-SEA total scores (*n* = 164)

Variables	Z-score equations
Facial Emotion Recognition Test (max = 35)	Raw Score – [30.344 + (−1.518 x Sex) + (0.185 x Education) + (−0.058 × Age)]/2.625
Mini-SEA total (max = 30)	Raw Score – [25.373 + (0.110 x Education) + (−0.035 x Age) + (−0.541 × Sex)]/1.985

Table 9 displays the results of the percentile analyses for both the long and short FPTs, whose scores were skewed. Percentiles were not stratified according to sociodemographic characteristics since none of these factors were found to be significantly correlated with the long and short-FPT scores. The table shows the scores corresponding to percentiles 1, 2, 5, 10, 15, 25, 35, 45, 50, 55, 65, 75, 85, 90, 95, 98, and 99. As per the guidelines of the American Academy of Clinical Neuropsychology (Guilmette et al., 2020), a score corresponding to a percentile rank of 24 or higher is considered within the normal range. Percentile ranks from 23 to 9 indicate a low average performance, while ranks from 8 to 2 suggest below-average performance, with scores below 2 classified as extremely low. For the control questions, more than 95% of participants in the sample scored 19 or 20 (out of 20) on the long FPT and, for the short-FPT, more than 99% of participants scored 9 or 10 (out of 10).

**Table 9:** Percentile ranks for the Faux Pas Test and the short Faux Pas Test (*n* = 168)

Percentile	Score on Faux Pas Test (/40)	Score on the short Faux Pas Test (/22)
1	20	8
2	22	11
5	25	13
10	26	14
15	28	15
25	30	18
35	31	19
45	32	19
50	32	20
55	33	20
65	35	20
75	36	21
85	37	21
90	38	21
95	39	22
98	40	22
99	40	22

A Microsoft Excel® spreadsheet has been created to facilitate the computation of Z-scores using the regression equations provided in Table 7 and an example of how to use the equation is presented in the discussion section. The spreadsheet also includes the percentile values for the FPT presented in Table 9. Interested clinicians and researchers may download the file from the journal website’s Supplementary Materials section or request it from the corresponding author.

## DISCUSSION

The objectives of this study were to establish the test–retest reliability and the known-group discriminant validity of the French-Quebec adaptation of the mini-SEA. Additionally, this study aimed to generate French-Quebec normative data for the mini-SEA, which are tailored to different socio-demographic variables in individuals aged 50 years and older. As social cognition is now recognized as one of the six primary core domains of cognition according to the DSM-5, and considering that social cognition is impaired in many neuropsychiatric, neurodevelopmental and neurological conditions as well as in neurodegenerative diseases, numerous authors have suggested that a comprehensive investigation of social cognition abilities should become a standard component of the neuropsychological assessment of patients (Adenzato & Poletti, 2013; Henry et al., 2016). The findings of this study showed that the mini-SEA can effectively discriminates between cognitively healthy individuals and those living with MCI or AD. Moreover, the performance of cognitively healthy participants on the mini-SEA remained stable over a period of approximately 3 to 4 months. Furthermore, the results indicated that socio-demographic variables were associated with some performance indices of the mini-SEA. Specifically, age, education, and sex were associated with performance on the mini-SEA (total score) and the FERT. However, sociodemographic characteristics of the participants were not associated with performance on the FPT, including its short version. For the mini-SEA total score as well as the FERT score, normative data were derived from regression equations to calculate Z-scores. For the FPT and its short version, normative data were produced using percentile ranks.

Based on the existing literature, it is consistent that the mini-SEA can distinguish cognitively healthy individuals from those with MCI or AD. Numerous studies have shown significant impairments in various aspects of social cognition among individuals with MCI or AD (Bora & Yener, 2017; Klein-Koerkamp et al., 2012; Schild et al., 2021), with more severe deficits observed in AD than in MCI (Spoletini et al., 2008; Yi et al., 2020). The findings of the current study align with the literature, as the mini-SEA, as well as the FPT (which measures ToM), revealed significantly better performance in cognitively healthy participants compared to those living with MCI or AD. There was minimal or no overlap in the mini-SEA total and FPT mean scores intervals between cognitively healthy participants and the MCI group, and between cognitively healthy participants and the AD group. However, it is worth noting that the FERT did not show significant differences between groups. The latter finding is at odds with the literature showing that people living with AD have significant impairments in facial emotion recognition compared to cognitively healthy individuals (Klein-Koerkamp et al., 2012; Setién-Suero et al., 2022; Strijkert et al., 2021). Considering that the participants included in the AD group were at a relatively early stage of the disease, it is possible to speculate that the cognitive deficits observed in this particular AD group were not severe enough to result in noticeable reductions in facial emotion recognition. Also, as demonstrated by Chuang et al. (2021), some emotions (such as joy) are recognized equally well by cognitively healthy individuals as well as those with AD or MCI, while other emotions (such as sadness) are significantly less accurately recognized in individuals with these conditions compared to cognitively healthy individuals. Therefore, it is plausible to hypothesize that comparing our three groups based solely on the total score on the FERT in this study, rather than examining the individual emotions separately, may have attenuated the possibility of detecting differences, if any. Additionally, several studies have demonstrated lower performance in facial emotion recognition among individuals with MCI compared to cognitively healthy individuals (Barbieri et al., 2022; Bora & Yener, 2017; Tsentidou et al., 2022). However, a recent systematic review by Morellini et al. (2022b) reported mixed findings regarding facial emotion recognition in MCI: while some studies reported significant differences compared to cognitively healthy individuals, others found no differences. In relation to the distinction between individuals with MCI and those living with AD, the present study did not reveal any significant differences between the two groups in terms of performance on the two subtests or the global mini-SEA performance. This finding aligns with the conclusions of Kessels et al. (2020), who stated that the magnitude of social cognition deficits between individuals with MCI and AD did not differ.

Based on the findings of the current study, the results indicate a large association between the two measurement times for both the mini-SEA and its subtests since the correlation coefficients are 0.50 or greater, as suggested by Cohen (1988). According to the Compendium of Neuropsychological Tests (Sherman et al., 2023), the FPT and FERT have minimum degree of test–retest reliability in cognitively healthy individuals, with coefficients above 0.60. However, the results for the complete battery fall slightly below this criterion. It is worth noting that test–retest reliability measured at longer intervals (such as 3–4 months in the current study), although more representative of clinical practice, tends to yield lower coefficients. Nevertheless, clinicians should bear in mind that mini-SEA’s performance scores can be somewhat sensitive to the passage of time. To determine the extent to which the stability of scores on the mini-SEA is sensitive to the passage of time, this parameter should be re-examined in the future with a shorter interval between the two administrations.

The results of the current study revealed positive associations between mini-SEA scores and the education level, indicating that higher education was linked to better performance. Additionally, male sex and advancing age showed marginal negative associations with performance on the mini-SEA. When examining the subtests individually, the FERT exhibited a positive association with education and negative associations with male sex and advancing age. This means that higher education levels are associated with better performance on the FERT, while being a male and getting older were associated with slightly poorer performance. However, for the long and short-FPT, no significant associations were found with age, sex, or education level. For the control questions used in the FPT, if the total score is 18 or less for the long FPT or of 8 or less in the short-FPT, clinicians should be cautious in interpreting the scores. The control questions serve as an internal validity check to confirm that the participant has understood the story, preventing confusion between comprehension and social cognition challenges. Potential indicators of invalid responses include significant confusion, reading difficulties, repetitive questions, or simulation-like responses.

The negative association between advancing age and performance on the mini-SEA (total score) and the FERT subtest is consistent with empirical evidence from several studies that have investigated the trajectory of social cognition during the aging process (Baksh et al., 2018; Cavallini et al., 2013; Fernandes et al., 2021). Indeed, previous studies support the notion that some aspects of social cognition tend to decline with advancing age. Specifically, research has shown that the recognition of negative emotions tends to be less accurate compared with positive emotions in older adults (Ferreira et al., 2021; Keightley et al., 2006; Kemp et al., 2012). This finding aligns with the data presented in Table 6, where average recognition scores for fear and sadness were lower compared with joy and surprise, for example. Regarding ToM, the existing scientific literature suggests that performance in affective ToM remains relatively stable during normal aging, while cognitive ToM tends to decline (Bottiroli et al., 2016; Reiter et al., 2017). It is plausible to think that the lack of an age effect in the long and short-FPT subtests could be attributed to the fact that the total scores combine questions related to both cognitive and affective ToM. Since cognitive and affective ToM may exhibit different patterns of change with advancing age, combining them in a single score could have attenuated the potential to detect an effect. Nonetheless, a previous French (France) validation and standardization study of the mini-SEA reported that age is a predictor of overall mini-SEA performance, and this variable also influenced the results of the two subtests when analyzed individually (Quesque et al., 2020). Our findings reinforce the influence of age on social cognition and highlight the importance of considering age-related effects when interpreting mini-SEA total score and scores on its subtests.

The positive association between education and performance on neuropsychological tests is well-established in the literature (Opdebeeck et al., 2016). Accordingly, the present study reported positive associations between higher level of education, on the one hand, and performance on the mini-SEA and the FERT, on the other hand. Previous studies have linked higher levels of education to better performance in emotion recognition tasks (de Souza et al., 2018; Dodich et al., 2014). However, it is noteworthy that Quesque et al. (2020), in their validation and standardization study of the mini-SEA in France, did not observe any effect of education on the results of the mini-SEA total score or its two subtests. This discrepancy is consistent with the lack of a significant association between education and performance on the long and short-FPT in the current study. In the past, there has been little research specifically examining the influence of education on ToM, and it has yielded conflicting results. Consequently, it is difficult to draw firm conclusions regarding the relationship between education and ToM performance. For instance, two psychometric studies on a Faux Pas test reported contradictory findings, with one study observing no effect of education on performance (Şandor & İşcen, 2023), while another study showed that participants with higher education levels performed better on the FPT compared to those with lower levels of education (Faísca et al., 2016). These inconsistencies highlight the need for further research to elucidate the relationship between education and ToM performance.

The negative associations between male sex, on the one hand, and mini-SEA and FERT performance, on the other, are also consistent with numerous studies, including the work of Olderbak et al. (2019) as well as meta-analyses of McClure (2000) and Thompson and Voyer (2014). The latter research works suggest that women generally have a slight advantage over men in decoding emotions in others, which is consistent with the results of the present study. There are fewer data on the influence of sex on ToM, and the consensus is unclear. The validation and standardization study of the mini-SEA conducted in France by Quesque et al. (2020) also found slightly higher scores for women on the mini-SEA. However, when the two subtests were analyzed separately, no significant sex effect was found on the FERT or the FPT scores. This last finding is consistent with the results of the current study, which also found no significant sex effect on the short or long FPT. Overall, the findings in the literature support the notion that there may be gender differences in social cognition, particularly in emotion decoding, with women often showing a slight advantage over men.

To illustrate the use of regression equations to obtain norms adjusted for different sociodemographic variables, let us consider a 72-year-old man who has completed a master’s degree (18 years of education) and achieved a total score of 21.16 out of 30 (29/40 on the FPT; 24/35 on the FERT) on the mini-SEA. By entering the data appropriately into the equation, he would obtain a Z-score of −1.6, which falls in the below average range according to the interpretation criteria of Guilmette et al. (2020). If this man had instead completed the equivalent of a high school diploma (11 years of education), his score of 21.16, corresponding to a Z-score of −1.2, would be indicating a performance in the low average range (Guilmette et al., 2020). By using the French normative data from Quesque et al. (2020), the 72-year-old man with 18 years of education, as well as the one with 11 years of education, would both have obtained a Z-score of −2.1, indicating extremely low performance. This comparison highlights a critical difference: applying the French (France) norms to their results on the mini-SEA could lead clinicians to conclude the presence of cognitive impairment, whereas using the French-Quebec norms provides a more nuanced interpretation. This underscores the importance of using local norms that are culturally fitted (Boone et al., 2007). Social cognition is a complex, multifactorial construct, which involves several low- and high-level cognitive processes. Therefore, several reasons may explain the observed differences between French and French-Quebec norms, including social norms that may vary from one culture to another, as well as the type of normative data used (calculating Z scores from mean and standard deviation versus using regression to obtain a Z score). In future studies, it may be important to examine the construct validity of the mini-SEA, but also how social cognition differs qualitatively from one country or culture to another and what explains these differences.

### Strengths

This study is the first to validate a social cognition instrument and to produce normative data in the French-Quebec population aged 50 years and older. Moreover, the development of a short version of the FPT and the establishment of norms for this version are additional strengths of this study. Clinicians are often constrained by time limitations in conducting assessments and have expressed the need for shorter tools to evaluate ToM. Therefore, the short-FPT standards in this study will allow clinicians to assess ToM more quickly and easily. Given the general rule that shorter tests have lower reliability than longer ones, the reliability of the proposed shorter version of the FPT cannot be assured. Another strength of this study is the use of comprehensive inclusion and exclusion criteria, making it possible to rule out the effects of factors or variables that could have potentially influenced performance on the mini-SEA and its subtests. Furthermore, the integration of a thorough neuropsychological evaluation for participants in the MCI or AD groups ensured appropriate matching based on their overall cognitive profile. The use of a normalization method based on regression equations for the total score of the mini-SEA and the FERT score is also a strength of the study, as this technique provides a continuously adapted interpretation of the results based on participants’ specific sociodemographic characteristics. Additionally, the study benefits from a standardized procedure used for both the administration and scoring of the mini-SEA, as well as the continuous training provided to research assistants involved in data collection. Regular and random audits were conducted to ensure accurate administration, which is critical for the mini-SEA as it includes open-ended questions that may require additional probing when necessary. An independent research assistant also conducted a double-check of the study’s data to verify data entry errors or aberrant scores in the database. This rigorous approach strengthens the robustness of the study by ensuring the reliability and accuracy of the collected data.

### Limitations

The primary limitation of this study is the use of a convenience sample. Consequently, the sample is more educated and contains a higher proportion of women than the general population. Additionally, the sample does not include individuals aged 90 years and older. Therefore, caution should be exercised when generalizing the normative data to underrepresented populations (e.g., individuals with low education levels) or populations not represented in the study (e.g., individuals aged 90 years and older). Another limitation of this study relates to the relatively low percentages of variance explained by the regression models used in the production of normative data. However, it is not surprising that sociodemographic variables do not have a greater impact on the model, considering that social cognition is influenced by many other factors (e.g., culture). Since social cognition is highly sensitive to culture, it would have been valuable to conduct a multicenter study to compare performance across different cities or regions in Quebec (e.g., urban vs rural areas, Indigenous populations vs non-Indigenous populations). Such an approach could have revealed possible differences in norms between different regions and within more culturally diverse populations. Finally, for this study a Z-score greater than −1.5 was used as the cutoff score for the MoCA to determine participant inclusion. However, current recommendations suggest using a cutoff of *Z* = −1.3 (Guilmette et al., 2020). Thus, a few below-average individuals in terms of global cognitive functioning were included in the study. Nevertheless, we conducted an analysis comparing the results of those participants with MoCA scores between −1.3 and − 1.5 (*n* = 3), and the performances were comparable to those obtained from the total normalization sample. The decision was thus made to maintain the cutoff score of −1,5 in order to maximize the sample size without compromising the interpretation of the results.

## CONCLUSION

In summary, this study presents psychometric properties and normative data for the mini-SEA in the French-Quebec population aged 50 years and older. This represents a significant advance in the field and provides clinicians with a validated and standardized tool for assessing social cognition in middle-aged or older patients, which has not previously existed in the province of Quebec (Canada). Furthermore, this study demonstrates that performance on the mini-SEA can effectively discriminate between cognitively healthy individuals and those with neurocognitive disorders such as MCI or AD. This contribution enables improved interpretation and diagnosis of social cognition difficulties associated with aging, thereby facilitating better support and care for individuals affected by such challenges.

## Supplementary Material

Table_10_SUPPL_MATERIAL_acae051

Normative_data_mini-SEA_corrected_acae051
