# Correction to: Mini-SEA: Validity and Normative Data for the French-Quebec Population Aged 50 Years and Above

**DOI:** 10.1093/arclin/acaf070

**Published:** 2025-07-28

This is a correction to: Hannah Mulet-Perreault, Mariane Landry, Robert Jr Laforce, Joël Macoir, Carol Hudon, Mini-SEA: Validity and Normative Data for the French-Quebec Population Aged 50 Years and Above, *Archives of Clinical Neuropsychology*, Volume 40, Issue 3, May 2025, Pages 694–707, https://doi.org/10.1093/arclin/acae051

There was an error detected in the creation of the regression equations, making them unsuitable for clinicians to use in interpreting the originally-published Mini-SEA results. Specifically, one number, the square root of the mean square residual, in both equations was incorrect. By correcting this, the interpretation of Mini-SEA results will be accurate. There are corrections made to Tables 7 and 8, and correspondingly within the supplementary file, to facilitate the use of the regression equation for clinicians. The example illustrating the use of the regression equation on page 10 of the **Discussion** has also been revised to ensure consistency with the corrected equation.

Table 7 is emended to read:

**Table 7:** Coefficients for multiple linear regression analyses for the Facial Emotion Recognition Test (*n* = 167) and Mini-SEA total scores (*n* = 164)

Variables	*B*	**β**	*T*	*p*
**Facial Emotion Recognition Test** ^a^				
Age	−0.058	−0.168	−2.269	.025^*^
Education	0.185	0.195	2.552	.012^*^
Sex	−1.518	−0.266	−3.483	.001^*^
**Mini-SEA total** ^b^				
Age	−0.035	−0.140	−1.811	.072^*^^*^
Education	0.110	0.159	2.007	.046^*^
Sex	−0.541	−0.129	−1.622	.107^*^^*^

^a^Intercept = 30.344; Square root of the mean square residual = 2.625.

^b^Intercept = 25.373; Square root of the mean square residual = 1.985.

^*^Significant correlations coefficients.

^**^Marginally significant correlations coefficients.

instead of:

**Table 7:** Coefficients for multiple linear regression analyses for the Facial Emotion Recognition Test (*n* = 167) and Mini-SEA total scores (*n* = 164)

Variables	*B*	**β**	*T*	*p*
**Facial Emotion Recognition Test** ^a^				
Age	−0.058	−0.168	−2.269	.025^*^
Education	0.185	0.195	2.552	.012^*^
Sex	−1.518	−0.266	−3.483	.001^*^
**Mini-SEA total** ^b^				
Age	−0.035	−0.140	−1.811	.072^*^^*^
Education	0.110	0.159	2.007	.046^*^
Sex	−0.541	−0.129	−1.622	.107^*^^*^

^a^Intercept = 30.344; Square root of the mean square residual = 6.888.

^b^Intercept = 25.373; Square root of the mean square residual = 3.939.

^*^Significant correlations coefficients.

^**^Marginally significant correlations coefficients.

Table 8 is emended to read:

**Table 8:** Regression equation to calculate Z scores for each independent variables on the Facial Emotion Recognition Test (*n* = 167) and Mini-SEA total scores (*n* = 164)

Variables	Z-score equations
Facial Emotion Recognition Test (max = 35)	Raw Score – [30.344 + (−1.518 x Sex) + (0.185 x Education) + (−0.058 × Age)]/2.625
Mini-SEA total (max = 30)	Raw Score – [25.373 + (0.110 x Education) + (−0.035 x Age) + (−0.541 × Sex)]/1.985

instead of:

**Table 8:** Regression equation to calculate Z scores for each independent variables on the Facial Emotion Recognition Test (*n* = 167) and Mini-SEA total scores (*n* = 164)

Variables	Z-score equations
Facial Emotion Recognition Test (max = 35)	Raw Score – [30.344 + (−1.518 x Sex) + (0.185 x Education) + (−0.058 × Age)]/6.888
Mini-SEA total (max = 30)	Raw Score – [25.373 + (0.110 x Education) + (−0.035 x Age) + (−0.541 × Sex)]/3.939

The supplementary file is replaced with the emended version.

The emendations have been made to the article.

## Supplementary Material

Normative_data_mini-SEA_corrected_acaf070

