# Supplementary material for: Mini-SEA: Validity and Normative Data for the French-Quebec Population Aged 50 Years and Above
Source: Arch Clin Neuropsychol. 2024 Jun 25;40(3):694–707. doi: 10.1093/arclin/acae051 (PMC12034523; doi:10.1093/arclin/acae051)
Supplement: Table_10_SUPPL_MATERIAL_acae051 [file table_10_suppl_material_acae051.docx]

## SUPPLEMENTARY MATERIAL

**Table 10.** Comparison of participant’s performance on each story score of the long FPT for the cognitively healthy, mild cognitive impairment, and Alzheimer’s disease groups (*n*= 208).

| Stories, *mean (SD)* | Healthy  *n* = 168 | MCI  *n* = 20 | AD  *n* = 20 | *p* | H *vs.* MCI | H *vs.* AD | MCI *vs.* AD | |
| --- | --- | --- | --- | --- | --- | --- | --- | --- |
| **FPT’s stories scores** | | | | | | | |  |
| Story #1 (/2) | 1.9(0.4) | 2.0(0.0) | 1.6(0.8) | 0.011* | 0.392 | 0.005* | 0.007 | |
| Story #2 (/2) | 1.9(0.5) | 2.0(0.0) | 2.0(0.0) | 0.257 | - | - | - | |
| Story #3 (/6) | 4.9(0.9) | 4.2(1.4) | 4.2(1.4) | 0.005* | 0.018* | 0.012* | 0.916 | |
| Story #4 (/6) | 4.8(2.1) | 3.0(2.6) | 2.4(2.9) | < .001* | < .001* | < .001* | 0.928 | |
| Story #5 (/2) | 1.9(0.4) | 2.8(0.6) | 1.6(0.8) | 0.016* | 0.303 | 0.005* | 0.190 | |
| Story #6 (/2) | 2.0(0.3) | 1.9(0.4) | 1.8(0.6) | 0.095 | - | - | - | |
| Story #7 (/6) | 4.2(1.9) | 3.9(2.0) | 3.7(1.9) | 0.116 | - | - | - | |
| Story #8 (/6) | 5.2(0.8) | 4.5(1.5) | 3.6(2.0) | < .001* | 0.008* | < .001* | 0.277 | |
| Story #9 (/6) | 3.5(2.6) | 3.1(2.5) | 2.4(2.3) | 0.094 | - | - | - | |
| Story #10 (/2) | 2.0(0.2) | 1.8(0.6) | 1.9(0.4) | 0.008* | 0.011* | 0.173 | 0.249 | |

*Note*. *FPT* = Faux Pas Test; *H* = cognitively healthy; *MCI* = mild cognitive impairment; *AD* = Alzheimer’s Disease.

*Significant Kruskal-Wallis test, or Dunn post-hoc analysis for Kruskal-Wallis test.
